# IPET study: an FLT-PET window study to assess the activity of the steroid sulfatase inhibitor irosustat in early breast cancer

**DOI:** 10.1007/s10549-017-4427-x

**Published:** 2017-08-09

**Authors:** Carlo Palmieri, Richard Szydlo, Marie Miller, Laura Barker, Neva H. Patel, Hironobu Sasano, Tara Barwick, Henry Tam, Dimitri Hadjiminas, Jasmin Lee, Abeer Shaaban, Hanna Nicholas, R. Charles Coombes, Laura M. Kenny

**Affiliations:** 1 Department of Molecular and Clinical Cancer Medicine, University of Liverpool, Liverpool, L69 3BX UK; 2 Liverpool & Merseyside Breast Academic Unit, Royal Liverpool University Hospital, Liverpool, L7 8XP UK; 3 Academic Department of Medical Oncology, Clatterbridge Cancer Centre NHS Foundation Trust, Wirral, CH63 4JY UK; 4 Centre for Haematology, Imperial College London, London, W12 0NN UK; 5 Department of Surgery and Cancer, Imperial College London, London, W12 0NN UK; 6 Department of Medical Oncology, Imperial College Healthcare Trust, Fulham Palace Road, London, W6 8RF UK; 7 Radiological Sciences Unit and Department of Nuclear Medicine, Imperial College Healthcare NHS Trust, London, W6 8RF UK; 8 Department of Nuclear Medicine, Imperial College Healthcare NHS Trust, London, W6 8RF UK; 9 Department of Pathology, Tohoku University School of Medicine, Sendai, Japan; 10 Department of Radiology, Imperial College Healthcare Trust, Fulham Palace Road, London, W6 8RF UK; 11 Department of Surgery, Imperial College Healthcare Trust, Fulham Palace Road, London, W6 8RF UK; 12 Department of Pathology, Imperial College Healthcare Trust, Fulham Palace Road, London, W6 8RF UK; 13 Department of Histopathology, University Hospitals Birmingham NHS Foundation Trust, Birmingham, B15 2GW UK

**Keywords:** Breast Cancer, ER, FLT, PET, Ki67, Sulfatase, Irosustat

## Abstract

**Background:**

Steroid sulfatase (STS) is involved in oestrogen biosynthesis and irosustat is a first generation, irreversible steroid sulfatase inhibitor. A pre-surgical window-of-opportunity study with irosustat was undertaken in estrogen receptor-positive (ER+) breast cancer to assess the effect of irosustat on tumour cell proliferation as measured by 3′-deoxy-3′-[18F] fluorothymidine uptake measured by PET scanning (FLT-PET) and Ki67.

**Methods:**

Postmenopausal women with untreated ER+ early breast cancer were recruited, and imaged with FLT-PET at baseline and after at least 2 weeks treatment with irosustat, 40 mg once daily orally. The primary endpoint was changed in FLT uptake; secondary endpoints included safety and tolerability of irosustat, changes in tumoral Ki67 and steroidogenic enzymes expression and circulating steroid hormone levels.

**Results:**

Thirteen women were recruited, and ten started irosustat for 2 weeks, followed by repeat FLT-PET scans in eight. Defining response as decreases of ≥20% in standardized uptake value (SUV) or ≥30% in Ki, 1 (12.5% (95% CI 2–47%, *p* = 0.001)) and 3 (43% (95% CI 16–75%, *p* = <0.001) patients, respectively, responded. 6 out of 7 patients had a Ki67 reduction (range = −19.3 to 76.4%), and median percentage difference in Ki67 was 52.3% (*p* = 0.028). In one patient with a low baseline STS expression, a 19.7% increase in Ki67 was recorded. STS decreases were seen in tumours with high basal STS expression, significant decreases were also noted in aromatase, and 17β-hydroxysteroid dehydrogenase type 1 and 2. Irosustat was generally well tolerated with all adverse event CTCAE Grade ≤2.

**Conclusions:**

Irosustat resulted in a significant reduction in FLT uptake and Ki67, and is well tolerated. These data are the first demonstrating clinical activity of irosustat in early breast cancer. Baseline expression of STS may be a biomarker of sensitivity to irosustat.

**Electronic supplementary material:**

The online version of this article (doi:10.1007/s10549-017-4427-x) contains supplementary material, which is available to authorized users.

## Introduction

**Fig. 1:**
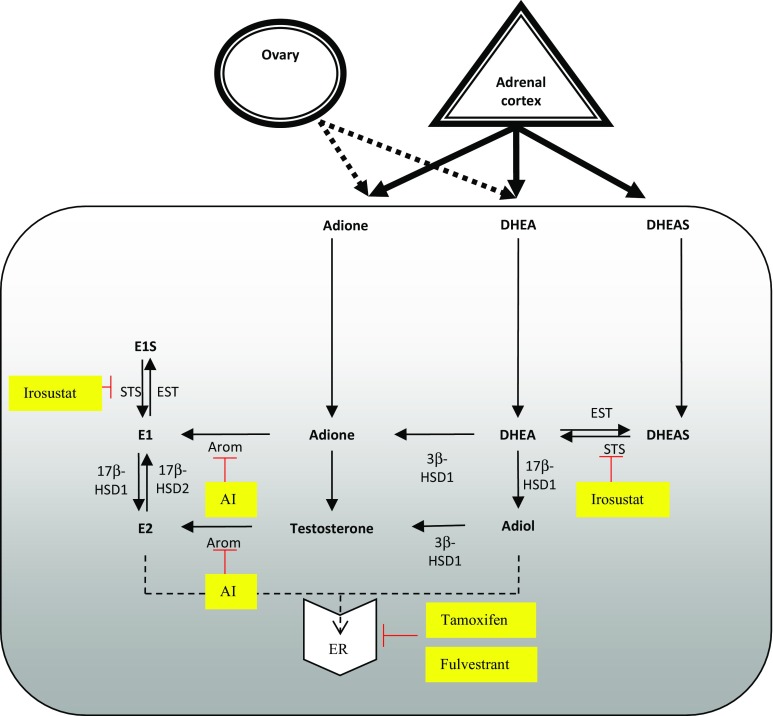
Source of steroidogenic hormones in postmenopausal women and sites of action of current endocrine therapies. *Adione* androstenedione, *Adiol* androstenediol, *AI* aromatase inhibitor, *Arom* aromatase, *E1* estrone, *E1S* estrone sulphate, *E2* estradiol, *ER* estrogen receptor, *EST* sulfotransferases, *STS* steroid sulfatase, *3β-HSD* 3β-hydroxysteroid dehydrogenase1/2, *17β-HSD type 1/2* 17β-hydroxysteroid dehydrogenase type1

The estrogen receptor (ER) is key therapeutic target in breast cancer, with over 70% of breast cancers expressing ER. Endocrine therapy (ET) is a key treatment modality in the treatment of such estrogen receptor-positive breast cancers and the disruption of the process of estrogen production by inhibition of the peripheral aromatisation (aromatase inhibitors) is a key form of ET [1], Fig. 1. The widespread use of adjuvant AIs has resulted in significant improvements in the overall survival of women with ER-positive early breast cancer [2]. However, resistance to ET is a major limitation to their use and efficacy, as well as a leading cause of relapse and death in breast cancer patients [3]. Additionally, aromatase represents one pathway for estrogen synthesis in the postmenopausal setting the other being via steroid sulfatase (STS). STS converts oestrone sulphate and dehydroepiandrosterone sulphate (DHEAS) to oestrone and DHEA, respectively. 17β-hydroxysteroid dehydrogenase type 1 (17β-HSD1) which is overexpressed in many breast cancers [4] converts oestrone to the biologically active estrogen, estradiol, and DHEA to androstenediol, which has potent estrogenic effects [5, 6], Fig. 1.

STS expression is higher in breast cancer than normal breast tissues, with 74% of breast cancers expressing STS [7, 8] and its expression at both mRNA and protein level has been associated with a poor clinical outcome [7, 8]. Conversely, expression of oestrogen sulfotransferase (EST) is inversely correlated with tumour size and lymph node status, as well as with a decreased risk of recurrence and improved overall survival [8]. Women progressing on third generation AI therapy have been shown to have a significantly higher serum DHEAS with estrogen suppressed below level of detection [9]. Furthermore, increase in intratumoral STS has been demonstrated following treatment with an AI indicating STS may be a possible compensatory and adaptive response to the depletion of intratumoural estrogen [10]. These data indicate that inhibition of STS could therefore offer an additional therapeutic strategy in the treatment of ER-positive breast cancer.

Irosustat (STX64; 667 Coumate; BN83495) is a first generation, orally active, irreversible STS inhibitor [11, 12]. It has been shown to cause regression of E1S-stimulated, nitrosomethylurea-induced mammary tumours in oophorectomized rats [13, 14]. Clinically, two phase I studies have been performed, [15, 16], and the optimal biological dose for phase II studies was determined to be 40 mg daily [16]. At this dose, no objective responses were seen, and the median time to progression was 10.1 (3.0–72.3) weeks [16]. Irosustat was well tolerated with no biochemical or hematologic toxicities related to irosustat reported [15, 16].

Inhibition of tumour proliferation is one of the key mechanisms of action of endocrine therapy. Changes in proliferation (as measured by Ki67) have been used to evaluate the biological activity of endocrine therapy in breast cancer in pre-operative window studies [17, 18] furthermore changes in Ki67 have been a validated intermediate endpoint for clinical trials [19]. The Immediate Preoperative Arimidex, Tamoxifen, or Combined with Tamoxifen (IMPACT) trial demonstrated that changes in Ki67 [19] mimicked the clinical outcomes in the larger equivalent adjuvant Arimidex, Tamoxifen, Alone or in Combination (ATAC) trial [20]. Therefore, evaluating changes in Ki67 levels in the context of pre-surgical studies could be used as an early marker of clinical efficacy and aid decision making around further development of agents concerned. The Ki67 labelling index, however, is subject to widespread variation between laboratories and studies; while criteria have been developed to standardize scoring to mitigate such variation, it remains problematic [21]. This is further complicated by the recognition of hotspots of proliferation within a single tumour that could be missed on biopsies; therefore, a biomarker that can capture tumour proliferation in its entirety may be preferable. One way forward is the in vivo imaging of proliferation by the use of PET imaging following injection of the thymidine analogue 3′-deoxy-3′-(18)F-fluorothymidine (FLT). FLT is a surrogate marker of proliferation as demonstrated by the correlation between breast tumour proliferation and FLT uptake as measured by semiquantitative (SUV) and model-derived parameters (Patlak Ki) [22, 23].

In the current study, we set out to test the hypothesis that inhibition of STS with irosustat for 2 weeks in patients with ER-positive breast cancer can result in a significant decrease in tumour proliferation as measured by FLT uptake and Ki67.

## Methods

### Study design

The IPET study (ClinicalTrials.gov identifier NCT01662726) was a single, open label phase II trial. Postmenopausal women with breast cancer were imaged with FLT-PET at baseline and after 2 weeks of therapy; changes in Ki67 were assessed in tumour biopsies around the same time-points as the PET scans. The study was approved by Dulwich Research Ethics Committee 12/LO/0269, and the UK Administration of Radioactive Substances Advisory Committee (ARSAC). The use of Irusostat was approved by the Medicines Health Regulatory Authority UK (EudraCT: 2011-005240-10). The study was done in accordance with the Declaration of Helsinki. All patients in the study were recruited from the medical oncology clinics attending Charing Cross Hospital (Imperial Healthcare NHS Trust, London). All patients provided written consent.

### Patient population

Eligible patients were postmenopausal with histologically confirmed ER-positive breast cancer (Allred ≥ 3), with primary tumours measuring ≥15 mm in longest diameter with no clinical evidence of metastatic disease. An Eastern Cooperative Oncology Group (ECOG) performance status of 0–2 and adequate haematological, renal, and liver function were required. Patients were excluded if they had used hormone replacement therapy or any other estrogen-containing medication or supplement or estrogen implants within 4 weeks of study entry. Patients were not enrolled if there was concomitant use of rifampicin and other CYP2C and 3A inducers, or systemic carbonic anhydrase inhibitors. Patients with any history of cardiac arrhythmias, or with risk/evidence of QTc prolongation were also excluded. Subjects unable to lie flat or fit into the PET/CT scanner could not enrol, similarly patients on occupational monitoring for radiation exposure were also excluded. The full inclusion and exclusion criteria are listed in the Supplementary Information.

#### Treatment

 Irosustat (Beaufour Ipsen Industrie Ltd) was administered as a 40-mg tablet taken once daily with water on an empty stomach, 30 min prior to breakfast. Treatment started the day after the baseline FLT-PET and was continued for a minimum of 2 weeks until the follow-up of FLT-PET scan. For those patients who have consented to a repeat tumour biopsy, treatment was extended to the day before the procedure.

### Trial assessments

Clinical assessments and toxicity reporting were performed at screening day 1, day 7, and day 14, and 7 days after the last dose of irosustat. Plasma was collected at day 0, day 7 and day 14 and formalin-fixed paraffin-embedded (FFPE) samples were collected of the primary tumour and the operative specimen following treatment. For those who entered prior to commencing neoadjuvant therapy, a repeat biopsy was optional. Data management was captured using the Inform^®^ electronic database system.

### Study endpoints

The primary endpoint was responsed as defined by changes in FLT uptake using PET following 2 weeks of irosustat treatment. Secondary endpoints were the safety and tolerability of irosustat as assessed by the collection of adverse events (AE) according to the Common Terminology Criteria for Adverse Events (NCI-CTCAE v 4.03) with relationship to study medication recorded, and there were coded using the Medical Dictionary for Regulatory Activities (MedDRA version 14.0) and changes in circulating steroid hormones. Exploratory translational endpoints included changes in proliferation as assessed by Ki67, the effects on intratumoral STS and the expression of steroidogenic enzymes.

#### PET imaging

FLT was manufactured according to a standard protocol as described previously [24]. All patients had dynamic imaging on a Siemens Biograph PET/CT scanner (64-slice; ICHNT, axial field of view (FOV),21.6 cm; transaxial, 60.5 cm) with the field of view centred on the tumour region of interest. CT was performed for attenuation correction. Baseline ^18^F-FLT PET/CT was performed within a week prior to start of irosustat treatment and the post-treatment PET/CT after 2 weeks of irosustat treatment.

Following intravenous injection of 220 MBq (±10%) of 18F-FLT patients were imaged for 66 min and 30 s, and the data were re-binned into the following time frames: 30 s × 1, 10 s × 10, 20 s × 4, 30 s × 4, 60 s × 7, 120 s × 4, 300 s × 3, 600 s × 3, 30 s × 1. Images were reconstructed using an iterative reconstruction algorithm (OSEM, 8 iterations, 21 subsets, 168 × 168, Gaussian filter, FWHM = 5 mm, scatter correction on and global scaling factor on). Image analysis was performed by an experienced operator on Hermes workstations (LK). Regions of interest were drawn manually on individual tumour lesions to create volumes of interest. One tumour VOI was identified for each patient, and the entire circumference of the tumour lesion was sampled. Average standardized uptake values (SUVs) and maximum SUVs (SUVmax) were corrected for the activity injected and body weight. For irreversible retention, a 2-cm volume was drawn on the left ventricle and this activity was used to derive an arterial input function. Metabolite analysis was performed by cartridge method, but the results were unsuccessful on repeatability assessments and therefore were not included in the data analysis.

#### Immunohistochemistry for steroidogenic enzymes

Immunohistochemistry of formalin-fixed and paraffin-embedded tissue sections was performed for the expression of four key steroidogenic enzymes involved in the biosynthesis pathway for oestrone and oestradiol: aromatase, steroid sulfatase (STS), estrogen sulfotransferase (EST), 17β-hydroxysteroid dehydrogenase (17β-HSD) type 1 and type 2 17β-HSD1, 17β-HSD2. Details of the methodology employed in this study have been previously published [8] and is described in Supplementary Materials and Methods.

 Immunostained slides were independently evaluated by two of the authors (GF, HS) blinded to patients’ clinical outcomes. The evaluation of the markers was performed by assessing the approximate percentage of immuno-positive areas (proportion score) and classifying the levels into four groups: 0 ≤ 1%, 1 = 1–25%, 2 = 26–50%, and 3 ≥ 50% immuno-positive cells for aromatase [25], or into three groups: 0 = no stained tumour cells, 1 = 1–50%, 2 ≥ 50% immuno-positive cells for STS, EST, and 17BHSD [8, 26], and the relative intensity of immuno-positive cells was classified as follows: 0 = no immunoreactivity, 1 = weak, 2 = moderate, and 3 = strong immunoreactivity. The total score was the addition of the proportion score and the relative immunointensity score (PIS Score).

### Immunohistochemistry for Ki67

4-µmm sections were used after heat-mediated antigen retrieval and stained with the primary antibody (Rabbit monoclonal anti-Ki67 antibody Clone 30-9, Ventana Medical Systems, Tucson, AZ). KI67 results were recorded independently by two investigators who were blinded regarding treatment allocation and each other’s assessment. 500 invasive cancer cells were counted for Ki-67 analysis, and considered positive Ki-67 staining as any tumour cells showing strong nuclear positivity. Only tumour cells which were obviously epithelial and atypical were counted. Any other cells showing positivity were disregarded, i.e., stromal cells or lymphocytes. The proportion of Ki-67-positive tumour cells taken as a proportion of 500 cells per case.

#### Steroid hormone analysis

Steroidogenic hormone profiling was performed on day 0, 7, 14, and was performed by a central laboratory, Quest Diagnostics (New Jersey Nichols Institute, San Juan Capistrano, CA, USA). Androstenedione, oestrone sulphate, dehydroepiandrosterone sulphate (DHEAS), dehydroepiandrosterone (DHEA), androstenediol, and testosterone were quantitated using a TSQ Quantum Ultra (Thermo Fisher; San Jose, CA) triple quadrupole tandem mass spectrometry. While estrone and estradiol were detected and quantitated on negative ionization mode using a triple quadrupole tandem mass spectrometer with APCI source (TSQ Quantum Ultra, Thermo Fisher; San Jose, CA). The detailed methodology is detailed in the Supplementary Materials and Methods.

### Statistical considerations

The response rate for patients receiving irosustat was estimated to be 30%, and so the number of patients required to give a predicted proportion of 30% with a window of response of 10–50% and 95% power was calculated to be 20. In order to maintain the appropriate sample size number with a predicted 20% drop-out rate, the total number of patients thus required for the study was estimate to be 24.

Comparisons between outcome and steroid measurements at baseline, and time-points thereafter, were carried out using the Wilcoxon signed-rank test. Obtained response proportions were compared with estimated proportions of response of 1% using a calculated *Z*-score. *P* values <0.05 were deemed statistically significant.

## Results

**Fig. 2:**
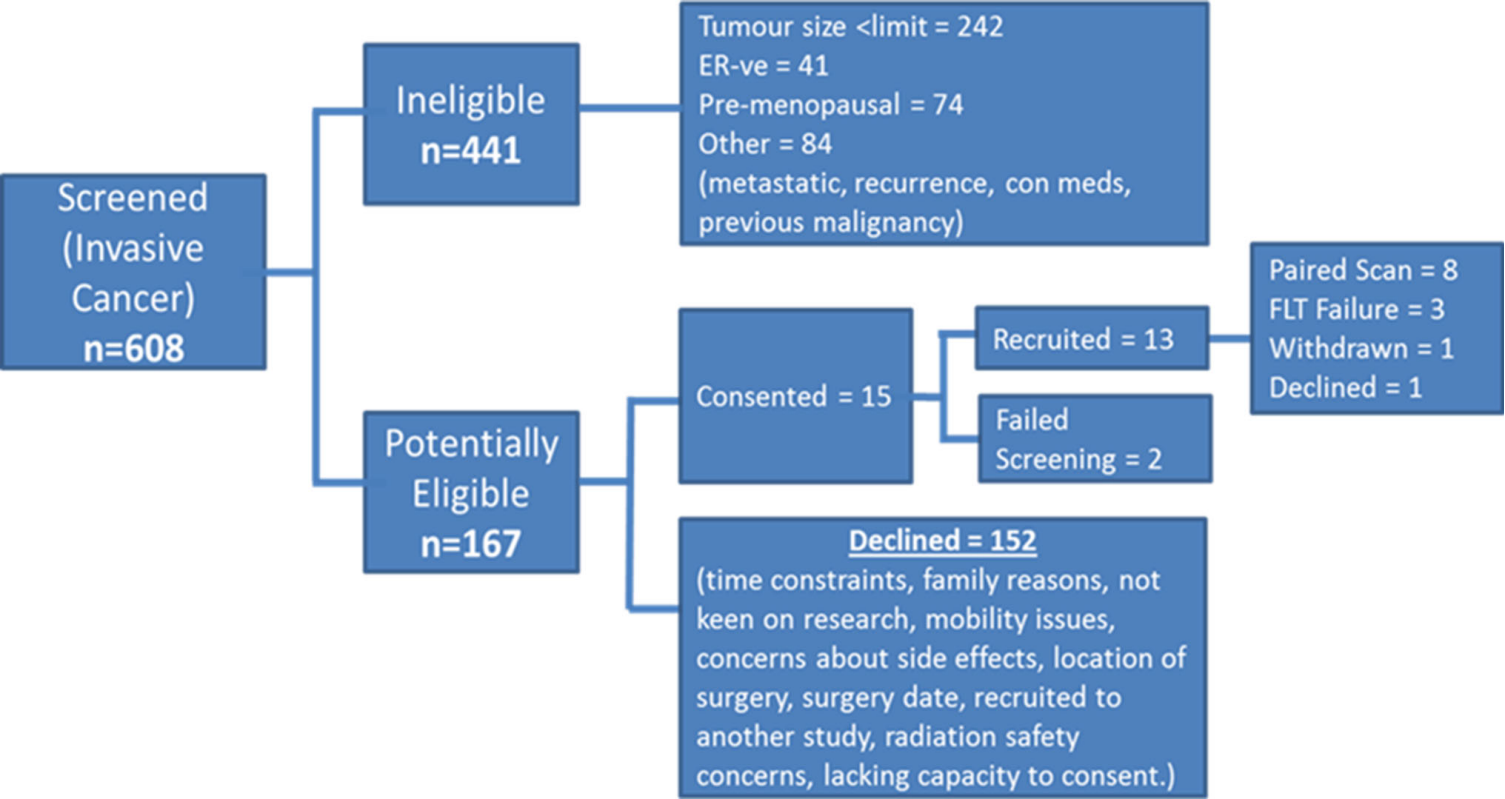
CONSORT diagram of the study describing the number of patients screened, eligible and ultimately recruited for the trial

Between November 2012 and November 2014–15 patients were recruited into the study and there were two screening failures. Of the 13 patients recruited into the study, there were two baseline FLT production failures, one declined on day 1 to enter, another was withdrawn after 14 days due to an abnormal ECG and in one case an FLT production failure prevented the on treatment PET therefore 8 paired FLT-PET scans were performed (Fig. 2). Paired translational samples were available in 7 of the 13 cases, on treatment samples were not available in two cases who went onto received neoadjuvant chemotherapy and in the case withdrawn because of ECG abnormality. However, a post-treatment sample was available in the case where there was FLT production failed at the second scan. Recruitment was terminated early due to the slow rate of accrual.

### Baseline characteristics and treatment compliance

The baseline clinicopathological information on all the patients both by intention to treat and per protocol are detailed in Table 1. In the per protocol analysis, all patients entered had strongly ER-positive tumours (Allred ≥ 7), 62% were strongly PgR positive (Allred ≥ 7) and 50% were HER2 positive (Table 1). Compliance with the study medication was good, with a median compliance rate of 100% (range 85.7–100%).

**Table 1:** Baseline clinicopathological features

	Per protocol (*N* = 8)	Intention to treat (*N* = 10)
	Median (range) or *N* (%)	Median (range) or *N* (%)
Age (year)	65.5 (52–79)	66.5 (52–82)
BMI (kg/m^2^)	26.3 (20.6–35.7)	26.3 (20.5–35.3)
Ethnicity		
White	4 (50%)	5 (50%)
Other White	4 (50%)	5 (50%)
Tumour size (mm) by USS	21 (17–48)**	21 (15–48)*
Hormone status		
ER (Allred score)		
7	1 (12.5%)	1 (10%)
8	7 (87.5%)	9 (90%)
PgR (Allred score)		
3	1 (12.5%)	1 (10%)
5	1 (12.5%)	1 (10%)
6	1 (12.5%)	1 (10%)
7	2 (25%)	4 (40%)
8	3 (37.5%)	3 (30%)
HER2		
Positive	4 (50%)	4 (50%)
Negative	4 (50%)	6 (60%)

*BMI* Body mass index, *ER* estrogen receptor, *mm* millimetres, *PgR* progesterone receptor

#### FLT-PET tumour response

Defining response as a decrease of ≥20% in SUV, 50% (95% CI 22–78%) of patients had a reduction in average SUV, and 1 patient (12.5% of total 95% CI 2–47%) responded to treatment based on average SUV.

75% (95% CI 41–93%) patients had a reduction in SUVmax. 71% (95% CI 36–92%) had a reduction in Ki, assuming a >30% reduction in Ki can be classed a meaningful response, 43% (95% CI 16–75%) (*n* = 3, patient no: 2, 6, 9) responded to treatment based on Ki, Supplmentary Table 1 demonstrates data per patient.

**Fig. 3:**
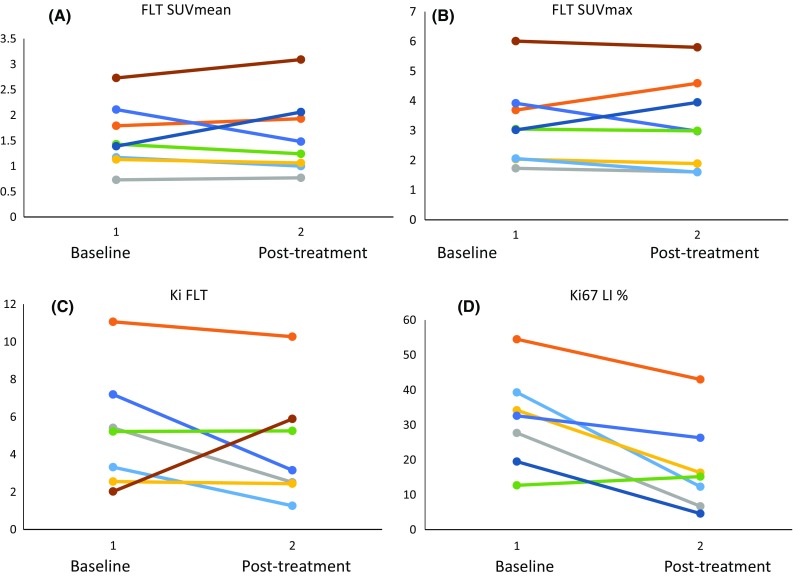
Graphs showing the changes for **a** SUVmean, **b** SUVmax, **c** Ki, and **d** Ki-67 labelling index for individual patients between baseline and after 14 days of irusostat

**Fig. 4:**
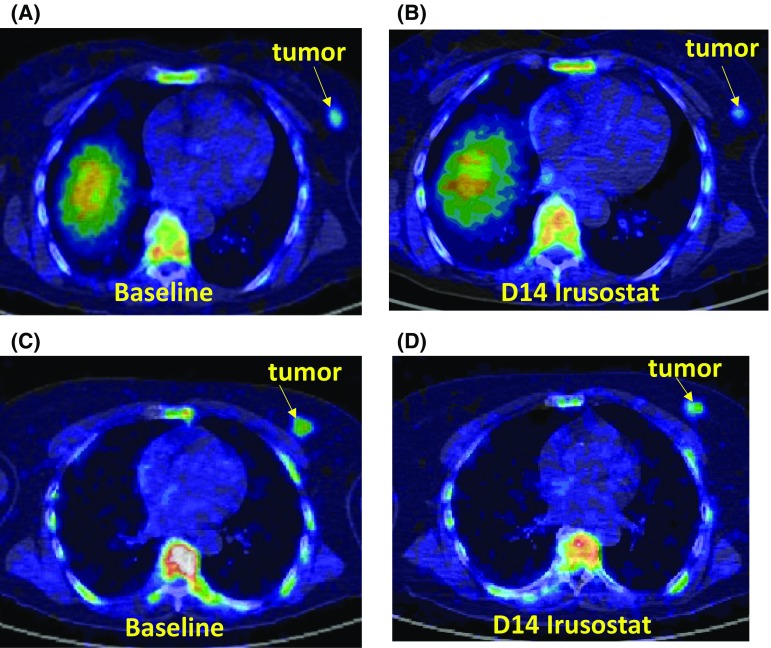
FLT-PET Images, *top panels* **a** and **b** show patient 009 an irusostat responder, SUVmax decreased from 3.92 to 2.98 and Ki also decreased from 7.2 to 3.1, the *bottom panels* (**c**, **d**) show a non-responder, SUVmax increased from 3.69 to 4.59 and Ki was not changed significantly (11.2 to 10.3)

Amongst the Ki responders, patient 2 had a 68.7% drop in Ki67, while patient 9 had a fall in both parameters (29.9% drop in SUV and a 56.2% fall in Ki); this patient also had a 52.3% drop in Ki-67. Unfortunately, patient 6 went on to receive neoadjuvant chemotherapy and a post-irusostat treatment sample was not available for comparison with FLT. 7 of the 8 patients were evaluated for changes in Ki as in patient 11 the Patlak model fitted poorly. The absolute changes in SUV and Ki, are shown in Fig. 3 and Supplmentary Table 1. Changes for SUV and SUVmax were similar. Pre- and post-treatment images of an FLT-responder and FLT non-responders are shown in Fig. 4.

### Changes in proliferation as measured by Ki67

Two independent pathologists scored the Ki67 labelling index in the seven paired cases. There was good concordance between both reporters with no difference by paired *t* test (*p* = 0.52) or by paired non-parametric test (*p* = 0.55) (Supplmentary Fig. 1), the absolute changes in Ki67 are shown in Supplmentary Table 2. Six patients (85%) had a reduction in Ki67, with a median percentage difference of 52.3% (range −19.3 to 76.4%), (Supplmentary Table 2), this reduction was statistically significant (*p* = 0.028). In only one case (patient 12), there was a 19.7% increase in Ki67, and interestingly in this case also had a rise in FLT-SUV (from 2.73 to 3.09) and FLT-Ki (from 2.02 to 5.89). Patient 7 had a large fall in Ki67 (75.8%) that was not associated with a significant fall in FLT uptake, while patient 14 who had a large decrease in Ki67 (76.4%) did not undergo an on treatment FLT-PET scan due to a production failure.

### Changes in expression of intratumoral steroidogenic enzymes

**Fig. 5:**
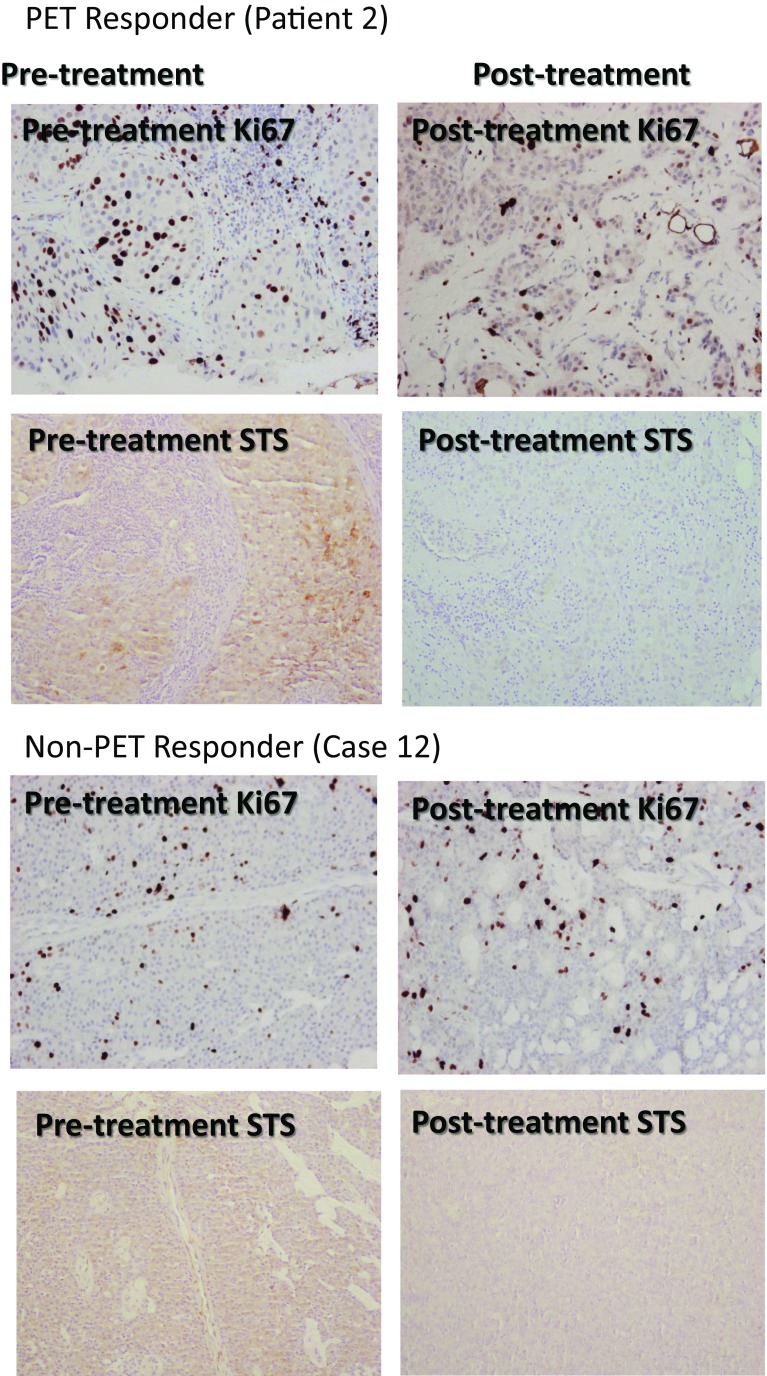
Immunohistochemistry of Ki67 and STS pre- and post-Irusostat changes in a FLT-PET responder and non-responder

Assessment of the expression of the steroidogenic enzymes aromatase, STS, EST, 17β-HSD type 1 and type 2 was undertaken in the 7 paired cases. This revealed that 57% had high STS expression at baseline (scores ≥ 4) and these cases all had notable decreases in STS after treatment with irosustat (Supplementary Table 3). Of note, in the one case where there was an increase in Ki67, this case had a low STS expression on both the baseline and end of treatment core biopsy. Figure 5a shows a PET responder (patient 2) and the corresponding reduction in Ki67 and STS following treatment, while Fig. 5b shows a PET non-responder with weak STS immunostaining at baseline with no reduction in Ki67 (patient 12). If response is defined as any reduction in the proportion and relative immunointensity score then 71% (5 of 7) patients had a response in aromatase, 57% (4 of 7) had responses in STS, 57% (4 of 7) in 17 β-HSD2 and 29% (2/7) had a reduction in 17 β-HSD1, all these reductions are statistically significant *p* < 0.0001.

### Changes in circulating steroidogenic hormones

Circulating steroidogenic hormone levels were available on days 0, 7, and 14; and at the available at the time of safety follow-up, and are shown in Table 2 and Supplementary Fig. 2. Significant reductions were seen in circulating estrone at day 14 which decreased by a median of 14 ng/dL (*p* = 0.017), and DHEA by a median of 82 ng/dL (*p* = 0.043), while there was a significant rise in DHEAS, median 62 ng/dL (*p* = 0.043). Changes in DHEAS/DHEA ratio, estrone sulphate, androstenedione, testosterone, estrone sulphate/oestrone ratio, and estradiol were not significant.

**Table 2:** Changes in circulating steroidogenic hormones following irosustat treatment

	Day 1 [*N* = 8] median (range)	Day 7 [*N* = 8] median (range)	Day 14 [*N* = 8] median (range)	Safety follow-up [*N* = 5] Median (range)
Oestradiol (pmol/L) ng/dL	9.5 (4–19)	8.5 (3–31)	7 (3–28)	5 (3–12)
Oestrone (ng/dL)	34.0 (22–62)	15.5 (9–36)	16 (9–41)	17 (9–23)
Androstenedione (nmol/L) ngIdL	55.0 (42–120)*	38 (16–61)	48 (7–97)	47 (21–81)
Oestrone Sulphate (pmol/L) ng/dL	376.5 (201–2001)	680 (148–1977)*	664.5 (172–2240)	261 (222–1114)
Dehydroepiandroste rone sulphate (DHEAS) (pmol/L) ng/dL	102 (30–221)**	101 (30–271)***	160 (45–394)***	72 (31–379)
Dehydroepiandroste rone (DHEA) (pmol/L) ng/dL	179 (49–279)*	87 (40–216)	100 (75–169)	68 (49–156)
Androstenediol (pg/ml) ng/dL A	NA	NA	NA	NA
Testosterone (nmol/L) ng/dL	22 (9–37)***	11 (9–19)***	4 (4–22)^N = 3^	8 (5–18)

*DHEA* Dehydroepiandrosterone, *DHEAS* dehydroepiandrosterone sulphate

* N = 7, ** *N* = 6, *** *N* = 5, Any missing values are due to quantity of sample not sufficient

### Safety data

Irosustat was generally well tolerated in the 10 patients who commenced treatment in the study. Sixty-four adverse events (AEs) were reported all of which were either grade 1 (81.3%) or grade 2 (17.2%) with grade missing in one (Table 3). 62% (40) AEs were unrelated to Irosustat, and of the 12.5% (8) AEs that were definitely or probably related to study drug the majority of these were dry skin which is a recognized side effect of irosustat. There was one serious adverse event which was an allergic reaction to the dye used for sentinel lymph node biopsy and was unrelated to Irosustat.

**Table 3:** Treatment-emergent adverse events regardless of relationship to study drugs

Adverse event	Grade 1	Grade 2
Dry skin	6	1
Alk Phos Increase	4	0
Nasopharyngitis	3	0
Pruritus	2	0
Fatigue	2	0
Dizziness	3	0
ALT/AST increase	3	0
Abnormal ECG	0	2
Prolonged QT	1	0
Tachycardia	0	1
Constipation	1	0
Hot Flush	1	0
Mouth Ulceration	1	0
Nausea	1	0
Vomiting	1	0
Gastrooesphageal Reflux	1	0
Hypersensitivity	1	1
Nipple infection	1	0
Sore throat	1	0
Viral pharyngitis	1	0
Arthralgia	1	0
Back pain/spinal pain/muscle spasm	2	1
Spinal osteoarthritis	1	0
Musculoskeletal discomfort	0	1
Pain in extremity	1	1
Headache	1	0
Breast mass	1	1
Dyspnoea	1	0
Syncope	1	0
Skin discolouration	1	0
Uticaria	1	0
Erythema	1	0
Thrombocytosis	1	0
Glycosuria	0	1
Proteinura	1	0
Hypermagnesaemia	1	0
Hypernatraemia	1	0
Hyperphosphatemia	1	0
Vitamin D deficiency	1	0

## Discussion

This study provides initial proof-of-concept data that suggest that STS inhibition is capable of reducing tumour proliferation as measured by ^18^F-FLT-PET in vivo, and in tumour biopsies by changes in Ki-67 in a cohort of previously untreated patients with breast cancer. More patients had a response in Ki-67 than with ^18^F-FLT-PET. Previous window studies have investigated ^18^F-FDG for PET imaging, but are complicated by metabolic flare, where an average 28% increase in ^18^F-FDG uptake is reported after 10 days of initiating treatment with tamoxifen, and paradoxically tumour flare was associated with subsequent response, probably due to the initial estrogenic activity of tamoxifen [27]. The median baseline Ki-67 was much lower in that study compared to our cohort (15 vs 36%). Kurland et al. [28] have studied changes in FDG uptake in breast cancer patients undergoing treatment with aromatase inhibitors and found that the degree of flare was less at 2 weeks (no patient had an increase in FDG SUV >11%) and for 11 patients who had a decrease in FDG uptake >20%, there was a correlation with the post-treatment Ki-67 (i.e., all of these patients had a Ki-67 post-treatment value of ≤5%. Likewise for patients where the decline in FDG SUV was <20%, the associated Ki67 at follow-up was >5% [28]. Only one patient in our study had a post-treatment Ki67 of <5%, suggesting that perhaps a longer duration of treatment or combination strategies are warranted in future studies. Alternatively, a higher dose might be needed to ensure that peripheral and intratumoral STS is more effectively inhibited.

To avoid any possibility of tumour flare, FLT was chosen as the probe for this study, as it recognized as the most advanced probe in clinical studies for assessment of tumour proliferation [29] and replaces the 2-[11C] thymidine-PET studies which are intrinsically difficult due to the short half-life and complex analysis required [30, 31]. This is the first study that has used FLT to assess response to endocrine therapy. Other studies have investigated Fluoroestradiol (FES), and shown that selective estrogen receptor modulation or down-regulation with tamoxifen and fulvestrant respectively reduced uptake more than treatment with aromatase inhibitors [32]. Whilst radiolabelled substrate probes for hormone receptors have shown promise for imaging response to endocrine therapy, the progression to hormone independence and emergence of endocrine resistance with loss of ER expression is a widely recognized phenomenon in breast cancer, and has been reported to occur in up to 35% of cases [33]. Although PERCIST criteria have been developed for FDG response in breast cancer, and specify a 30% reduction in uptake is necessary for response, these are generally not applied to short window studies where 20% has been accepted in previous study as the cut-off for response [34]. The PERCIST criteria rely on SUVmax, whereas more recently tumour lesion glycolysis (TLG) which takes tumour volume into account was superior for predicting pathological response to chemotherapy [35].

There was a significant reduction in the proliferation marker Ki67 after 2 weeks of irosustat, with a similar magnitude to that reported with 14–21 days treatment with tamoxifen [17, 19, 36]. But less than the reduction reported after 14 days of treatment with an aromatase inhibitors [19] or the selective estrogen receptor downregulator, fulvestrant, respectively [37]. However, it should be noted that 50% of cases in IPET were HER2 positive, and this may have therefore impacted on the FLT-PET data and Ki67 changes reported here given it is known that HER2 gene amplification reduces the antiproliferative effects of endocrine therapy [38, 39]. Despite the possible impact of the HER2, the significant suppression of Ki67 with irosustat provides proof of concept that inhibition of STS can affect tumour proliferation and based on the limited number of cases is at least comparable to tamoxifen. A larger study with Ki67 as a primary endpoint is required to further investigate the antiproliferative effects of STS inhibition both with and without an aromatase inhibitor. The current study did not require STS expression for study entry but given that STS inhibition was not universal, that the only case to have an increase in Ki67 had a low STS expression and the targeted nature of irosustat it would seem appropriate to pre-select breast cancers for STS expression in any future studies.

Irosustat resulted in a significant reduction in oestrone and DHEA, with a significant rise in DHEAS, this is in keeping with the mechanism of action and the previously published data [15, 16]. While previous phase I studies have demonstrated significant reductions in estradiol, androstenedione and testosterone [15, 16], the current study did not and this is may reflect the number of paired samples available and/or the duration of the treatment. Based on the steroidogenic hormone changes within the current study, the antiproliferative effect reported within the current study is likely to be related to the changes in DHEA and the subsequent effect on Adiol, although insufficient plasma samples precluded the analysis of Adiol. DHEA can be converted by 17β-HSD1 to Adiol, although an androgen Adiol can bind to and cause proliferation of ER-positive breast cancer cells in an ER-dependent manner [5, 40]. With *In vivo* rodent models of carcinogen-induced mammary carcinomas have demonstrated the ability of Adiol to stimulate tumour growth, even in the presence of aromatase inhibitors, confirming that this hormone does not need to be further aromatized to reveal its estrogenic effects [6]. While STS inhibition prevented DHEAS-stimulated growth of MCF-7 breast cancer cells, an effect which was not reproduced by concurrent treatment with aromatase inhibitors [41], and serum DHEAS have been shown to be significantly higher in women who progressed on an AI [9]. Further larger studies are needed to explore the effects of irosustat both alone and in combination with an aromatase inhibitor on circulating steroidogenic hormones.

Despite the large number of patients screened for the trial, recruitment in this pre-surgical population was challenging, with 73% of possible cases deemed ineligible based on entry criteria while 27% declined involvement. The fact that recruitment was in a single centre study, which was also a regional breast screening unit is likely to have impacted on recruitment. The latter is reflected in the fact that the biggest reason for ineligibility was the small size of tumours (58% of ineligible case). There were three instances in which the production of FLT failed and therefore impacted on the number of patients able to contribute FLT-PET data, and highlights one of the issues with functional imaging studies. The centre involved also had not participated in a major way in the POETIC study [42], a national window study and this lack of involvement and experience by may have indirectly impacted on recruitment. The FLT-PET sub-study of POETIC had similar challenges and failed to successfully recruit and issues similar to those documented in IPET impacted on recruitment including tumour size, reluctance of patients to participate given no obvious benefit to them, ability to fit PET imaging into a 2-week window and limited number of centres (personal communication Prof Fiona Gilbert). Therefore, the problems seen within IPET and FLT-PET POETIC may reflect an intrinsic issues with the undertaking of functional imaging studies in the context of pre-operative window studies in breast cancer, particularly given the successful completion of window studies which have utilized as the primary endpoint changes in proliferation as measured by immunohistochemistry rather than a functional imaging endpoint [42, 43]. Therefore, careful thought must be given in future to the development and implementation of PET studies in the pre-operative window of opportunity setting.

In conclusion, IPET provides proof of concept that irosustat can have an antiproliferative effects in ER-positive untreated breast cancer as defined by both FLT-PET and changes in expression of Ki67. Further window studies of irosustat utilizing changes in expression of Ki67 as the primary endpoint are warranted to further explore its activity in untreated early breast cancer both with and without an aromatase inhibitor. Such studies should enrich for breast cancers which are HER2 negative and that express STS expression given these are likely to derive the greatest benefit.

## Electronic supplementary material

Below is the link to the electronic supplementary material.

Supplementary Information 1 (DOCX 15 kb)

Supplementary Materials and Methods 2 (DOCX 18 kb)

Supplementary Figures and Tables 3 (PPTX 755 kb)
