# Supplementary material for: IPET study: an FLT-PET window study to assess the activity of the steroid sulfatase inhibitor irosustat in early breast cancer
Source: Breast Cancer Res Treat. 2017 Aug 9;166(2):527–39. doi: 10.1007/s10549-017-4427-x (PMC5668341; doi:10.1007/s10549-017-4427-x)
Supplement: Supplementary file 1 — Supplementary Information 1 (DOCX 15 kb) [file 10549_2017_4427_MOESM1_ESM.docx]

**Supplementary Information**

**Inclusion Criteria**

Patients who meet all of the following inclusion criteria will be considered eligible for this study:

1. Written informed consent to participate in the trial

2. 18 years of age or older

3. Histologically confirmed ER +ve breast cancer (Allred ≥3)

4. Any HER2 status

5. Tumour measuring ≥15mm in longest diameter on either mammography, ultrasound (US) examination or magnetic resonance imaging (MRI)

6. Postmenopausal women as defined by any one of the following criteria:

Amenorrhoea >12 months at the time of diagnosis and an intact uterus OR,prior bilateral oophorectomy OR,

FSH levels within the postmenopausal range (as per local practice) in women aged <55years who have undergone hysterectomy OR,

FSH levels within the postmenopausal range (as per local practice) in women aged <55 years who have been on Hormone Replacement Therapy (HRT) within the last 12 months and are therefore not amenorrhoeic

7. Eastern Cooperative Oncology Group (ECOG) performance status 0, 1 or 2

8. Adequate bone marrow function defined by Hb≥10 g/dl, WBC≥3.0 x109, PLT≥100 x109/L. Adequate renal function defined by a serum creatinine ≤1.5 x ULN. Adequate liver function defined by total bilirubin ≤ 1.5 ULN (patients with Gilbert’s syndrome exempted), either ALT or AST ≤1.5 ULN and ALP ≤1.5 ULN

**Exclusion Criteria**

1. Locally advanced/inoperable breast cancer

2. Clinical evidence of metastatic disease

3. Diffuse or inflammatory tumours

4. Any history of invasive malignancy within 5 years of starting study treatment (other than adequately treated basal cell carcinoma or squamous cell carcinoma of the skin and cervical carcinoma in situ)

5. Evidence of bleeding diathesis and PTT and PT ≤ 1.5 x upper limit of normal

6. Concomitant use (defined as use within 4 weeks prior to entry) of HRT or any other oestrogen-containing medication or supplement (including vaginal oestrogens and phytoestrogens)

7. Previous use of oestrogen implants at ANY time.

8. Concomitant use of:

a. Rifampicin and other CYP2C and 3A inducers such as rifabutin, rifapentine, carbamazepine, phenobarbital, phenytoin and St. John’s Wort

b. Systemic carbonic anhydrase inhibitors

9. Any of the following cardiac criteria:

a. Mean resting corrected QT interval (QTcf) >450 ms, as calculated by frederica’s formula, obtained from 3 electrocardiograms (ECGs)

b. Any clinically important abnormalities in rhythm, conduction or morphology of resting ECG e.g. complete left bundle branch block, third degree heart block

c. Any factors that increase the risk of QTc prolongation or risk of arrhythmic events such as heart failure, hypokalaemia, congenital long QT syndrome, family history of long QT syndrome or unexplained sudden death under 40 years of age or any concomitant medication known to prolong the QT interval

10. Uncontrolled abnormalities of serum potassium, sodium, calcium or magnesium levels

11. Evidence of uncontrolled active infection

12. Evidence of significant medical condition or laboratory finding which, in the opinion of the investigator, makes it undesirable for the patient to participate in the trial

13. Subjects unable to lie flat or fit into the scanner

14. Patients on occupational monitoring for radiation exposure
