# Supplementary material for: IPET study: an FLT-PET window study to assess the activity of the steroid sulfatase inhibitor irosustat in early breast cancer
Source: Breast Cancer Res Treat. 2017 Aug 9;166(2):527–39. doi: 10.1007/s10549-017-4427-x (PMC5668341; doi:10.1007/s10549-017-4427-x)
Supplement: Supplementary file 2 — Supplementary Materials and Methods 2 (DOCX 18 kb) [file 10549_2017_4427_MOESM2_ESM.docx]

**Supplementary Materials and Methods**

**Collection and storage of plasma samples for steroid hormone profiling**

8ml of blood were collected at each of the described time points into K2 EDTA tubes and processed within an hour of venesection and the plasma was separated by centrifugation at 850 g for 10 min. The plasma was taken from the upper phase, leaving 3mm undisturbed above the buffy layer, aliquoted into sterile tubes and stored at −80 °C.

**Immunohistochemistry for steroidogenic enzymes**

4-µm sections were used with tissues deparaffinized in xylene for 10 min, three times, followed by hydration of the tissue in progressively dilute solutions of ethanol (100%, 95%, 70% and 50%) for 2 min each. The slides were then immersed in tap water for 5 min. In the case of EST and 17BHSD1, the slides were retrieved by heating (500W microwave, 20 min) in buffer pH9.0 (Histofine antigen retrieval solution, Nichirei) and 10 mM citric acid buffer pH6.0 respectively. These slides were then allowed to cool to room temperature for 20 min on ice and washed twice in phosphate buffer saline (PBS). All the slides were then incubated for 30 min at room temperature in a humidified chamber with the blocking solution commensurate with the relevant primary antibody and detection kit (Histofine SAB-PO kit; Nichirei, Tokyo, Japan). Slides were then incubated with primary antibodies (anti-aromatase 677 Novartis, 1:500; STS KM1049 Kyowa Medix, 1:100; EST anti SULT1E1 Sigma, 1:100; 17BHSD1 2E5 Abnova, 1:400; 17BHSD2 Proteintech, 1:200). Following overnight at 4°C incubation, the slides were then washed three times in PBS. Endogenous peroxidase activity was blocked using absolute methanol and 0.3% H2O2 (30 min at room temperature), and subsequently washed in PBS. Later on, the slides were incubated with the relevant Histofine SAB-PO kit secondary antibody for 30 min at room temperature and washed three times in PBS. Staining was visualized using streptavidin-biotin-peroxidase for 30 min at room temperature followed by exposure to a chromogen solution consisting of 3,3′-diaminobenzidine (DAB) solution (1 mM DAB, 50 mM Tris–HCl buffer pH7.6, and 0.006% H2O2) and counterstained with hematoxylin. For aromatase, the specimens were visualized using liquid DAB and substrate chromogen system (Dako). Previously verified positive controls consisting of placenta known to contain the protein of interest were included for quality purposes.

**Steroidogenic Hormone Profiling**: **Comprehensive steroid panel**

**Materials**

Both the extraction columns and analytical columns (we purchased from Thermo Scientific. The mobile phase consists of Ultrapure water and Methanol (both LCMS grade quality from Honeywell).

**Reagents**

11-Deoxycortisol, Corticosterone, Cortisone, Deoxycorticosterone were purchased from Sigma-Aldrich Inc. (St. Louis, MO). 17-Hydroxyprogesterone, 17-Hydroxypregnenolone, Androstenedione, DHEA, Pregnenolone, Testosterone were purchased from Steraloids, Inc. (Newport, RI). 18-Hydroxycorticosterone and Cortisone-[2,2,4,6,6,9,12,12-H] were purchased from Isosciences (King of Prussia, PA). Cortisol and progesterone were purchased from U.S. Pharmacopeia (Rockville, MD). 17α-Hydroxyprogesterone-d8 (4-Pregnen-17α-ol-3.20-dione-2,2,4,6,6,21,21,21-d8) and Pregnenolone-2,2,3,4,4,6-d6 – were purchased from CDN Isotopes (Quebec, Canada).

Steroid-free, double charcoal-stripped sera was purchased from Golden West Biologicals (Temecula, CA) and was tested for detectable steroids before use. All other chemicals were reagent grade or better and were purchased from commercial sources.

**Methodology**

The sample was prepared by acidifying 100 µL of serum with 20% formic acid containing the three internal standards to break up the ionic interaction from the carrier protein to release the analytes without precipitating. After vigorous mixing, the samples were incubated at room temperature for 15 to 20 minutes prior to being placed in the refrigerated autosampler for injection.

The Aria TLX System (Thermo Electron - Franklin, MA) injected the prepared sample and is loaded onto an extraction column at high flow rate. This created turbulence inside the column, which allowed the steroids to bind to the large particles of the extraction column, while protein and other debris freely flowed through and were discarded. Following the loading step, the flow is reversed and the sample is eluted off the extraction column and transferred to a reverse-phase C8 analytical column. A binary HPLC gradient is applied to the column resulting in the separation of the 13 steroids and 3 internal standards from each other and their metabolites.

The steroids were quantitated using a TSQ Quantum Ultra (Thermo Fisher; San Jose, CA) triple quadrupole tandem mass spectrometer. The tandem mass spectrometer permits the isolation of the parent compound within ± 0.5 m/z within the first quadrupole (Q1). In the second quadrupole (Q2), the parent ions collide with an inert gas (argon) to generate daughter ions which are selected in the third quadrupole (Q3). Inclusion of deuterated steroids such as cortisone, 17-hydroxyprogesterone and 17-hydroxypregnenolone as internal standards enabled absolute quantitation of the steroids by correcting for procedural losses or ion suppression caused by matrix effects in the atmospheric pressure chemical ionization (APCI) process.

**Oestrone and Oestradiol**

**Materials**

The extraction columns (Cyclone P 50 x 1.0mm) were purchased from Thermo Scientific. and analytical columns (Synergi Polar-RP, 4µm 150x2.0 mm were purchased from Phenomenex.

The mobile phase consists of Ultrapure water and Methanol (both LCMS grade quality from Honeywell).

**Methodology**

The sample was prepared by adding 300 µL of 30% aqueous ethanol solution and 50 µL of the internal standard solution (which consists of the deuterated estradiol in methanol) to 200 µL of patient serum. The ethanol solution is used to break up the ionic interaction from the carrier protein to release the analyte without precipitating. After vigorous mixing, the samples were incubated at room temperature for 15 to 20 minutes prior to being placed in the refrigerated autosampler for injection.

The Aria TLX System (Thermo Electron - Franklin, MA) injected the prepared sample and is loaded onto an extraction column at high flow rate. This created turbulence inside the column, which allowed the steroids to bind to the large particles of the extraction column, while protein and other debris freely flowed through and were discarded. Following the loading step, the flow is reversed and the sample is eluted off the extraction column and transferred to a reverse-phase ether-linked phenyl analytical column. A binary HPLC gradient is applied to the column resulting in the separation of estradiol to its metabolites.

Estradiol was detected and quantitated on negative ionization mode using a triple quadrupole tandem mass spectrometer with APCI source (TSQ Quantum Ultra, Thermo Fisher; San Jose, CA).
