# Supplementary material for: IPET study: an FLT-PET window study to assess the activity of the steroid sulfatase inhibitor irosustat in early breast cancer
Source: Breast Cancer Res Treat. 2017 Aug 9;166(2):527–39. doi: 10.1007/s10549-017-4427-x (PMC5668341; doi:10.1007/s10549-017-4427-x)
Supplement: Supplementary file 3 — Supplementary Figures and Tables 3 (PPTX 755 kb) [file 10549_2017_4427_MOESM3_ESM.pptx]

## Slide 1
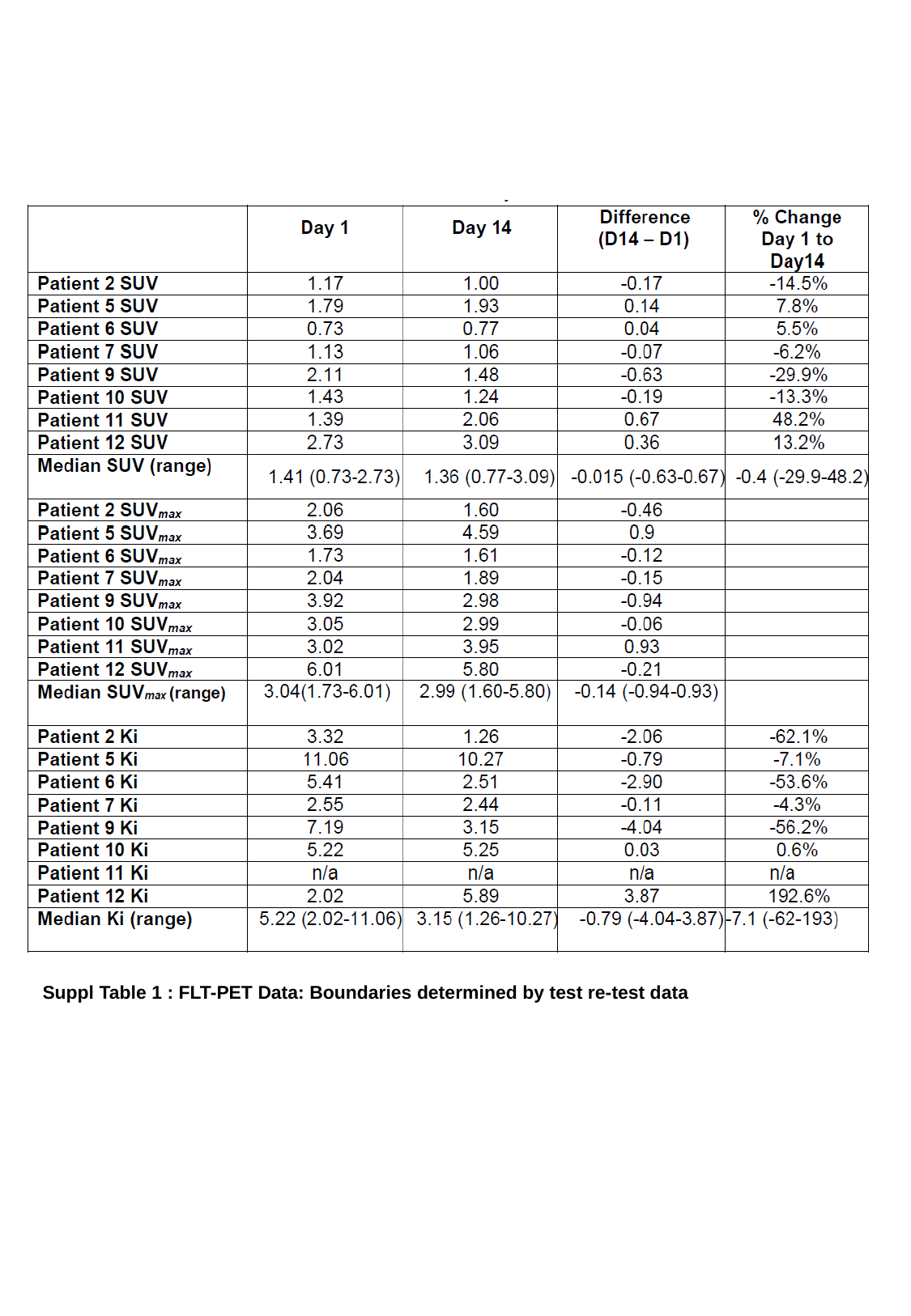

Suppl Table 1 : FLT-PET Data: Boundaries determined by test re-test data

## Slide 2
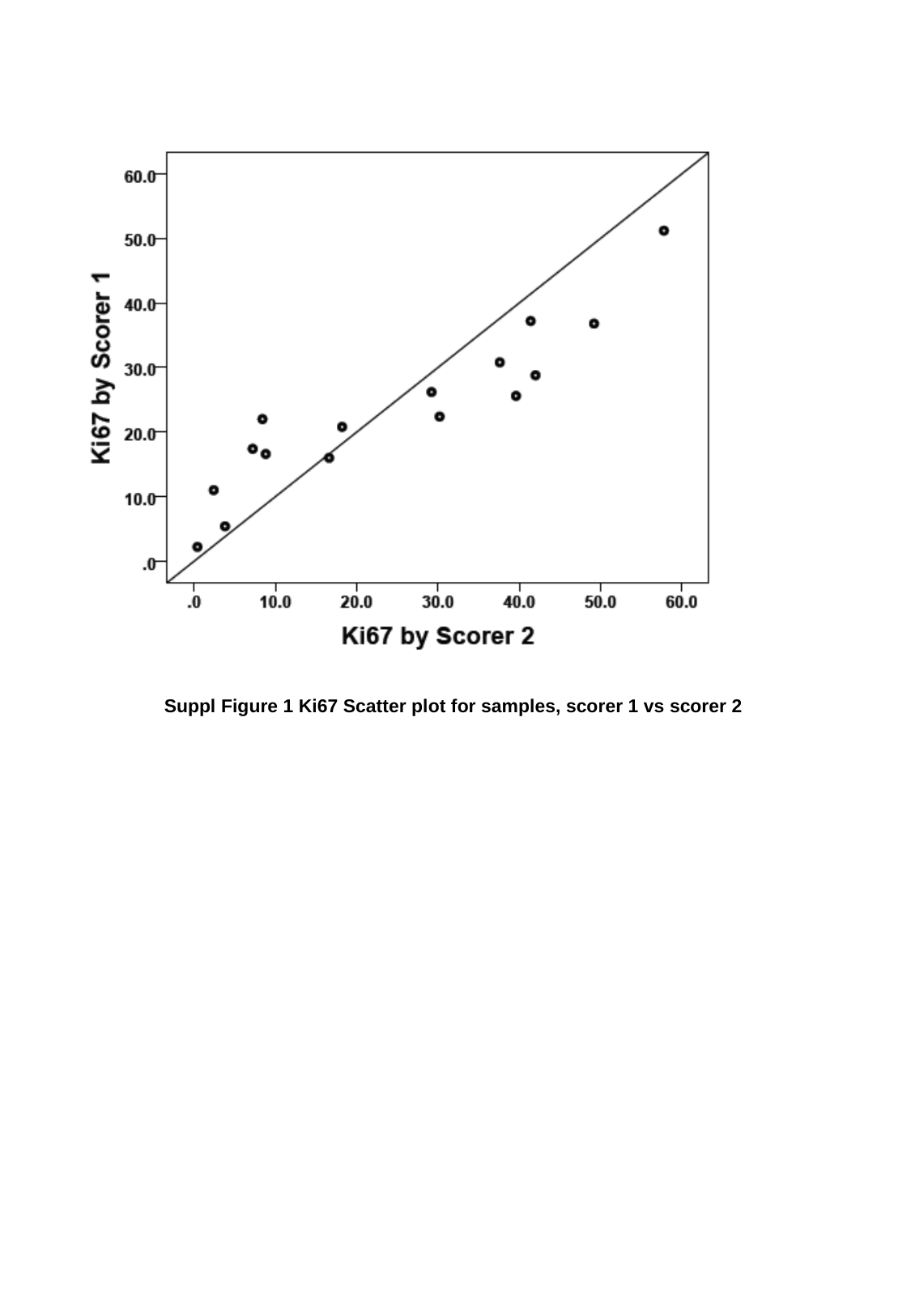

Suppl Figure 1 Ki67 Scatter plot for samples, scorer 1 vs scorer 2

## Slide 3
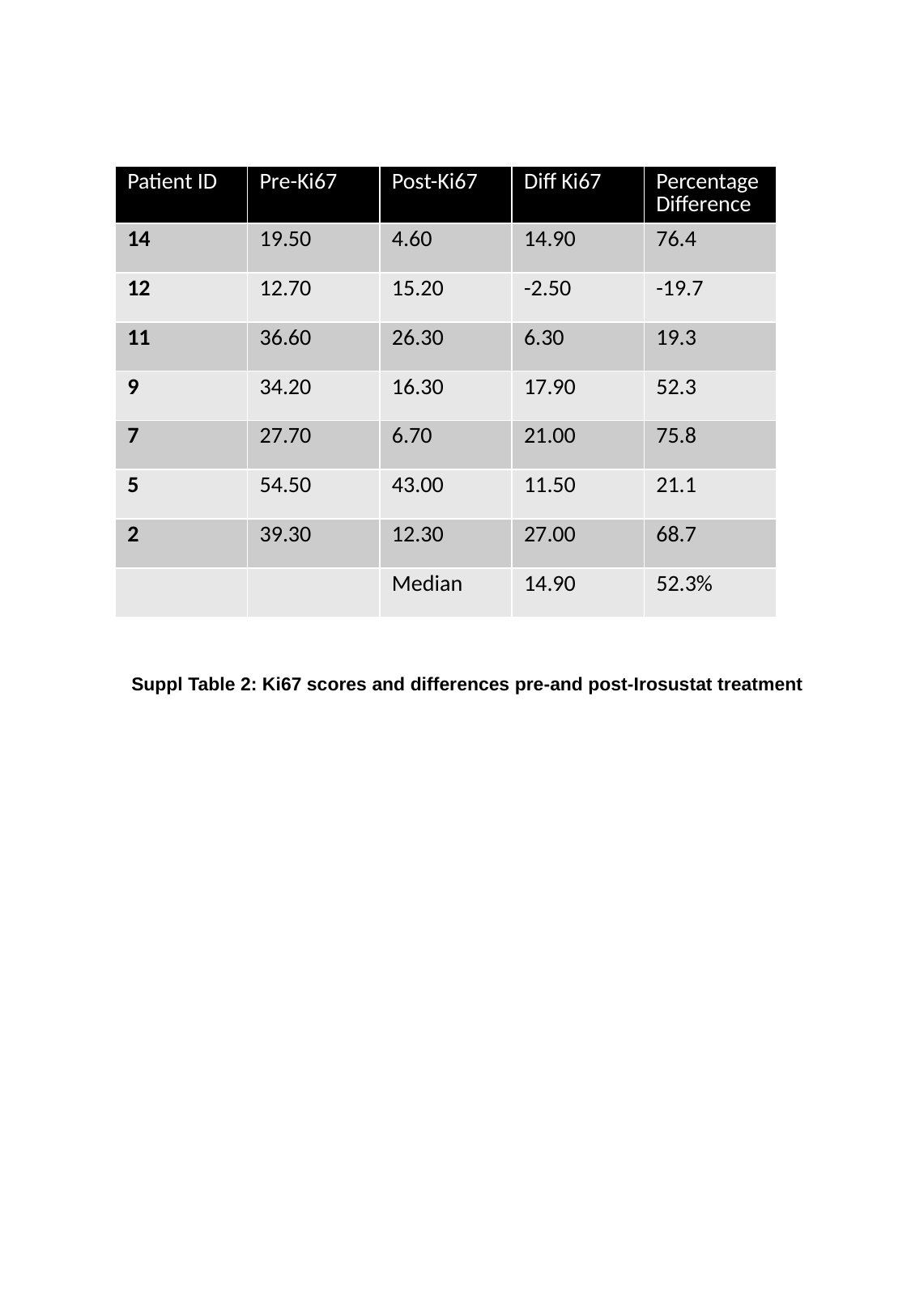

| Patient ID | Pre-Ki67 | Post-Ki67 | Diff Ki67 | Percentage Difference |
| --- | --- | --- | --- | --- |
| 14 | 19.50 | 4.60 | 14.90 | 76.4 |
| 12 | 12.70 | 15.20 | -2.50 | -19.7 |
| 11 | 36.60 | 26.30 | 6.30 | 19.3 |
| 9 | 34.20 | 16.30 | 17.90 | 52.3 |
| 7 | 27.70 | 6.70 | 21.00 | 75.8 |
| 5 | 54.50 | 43.00 | 11.50 | 21.1 |
| 2 | 39.30 | 12.30 | 27.00 | 68.7 |
| | | Median | 14.90 | 52.3% |
Suppl Table 2: Ki67 scores and differences pre-and post-Irosustat treatment

## Slide 4
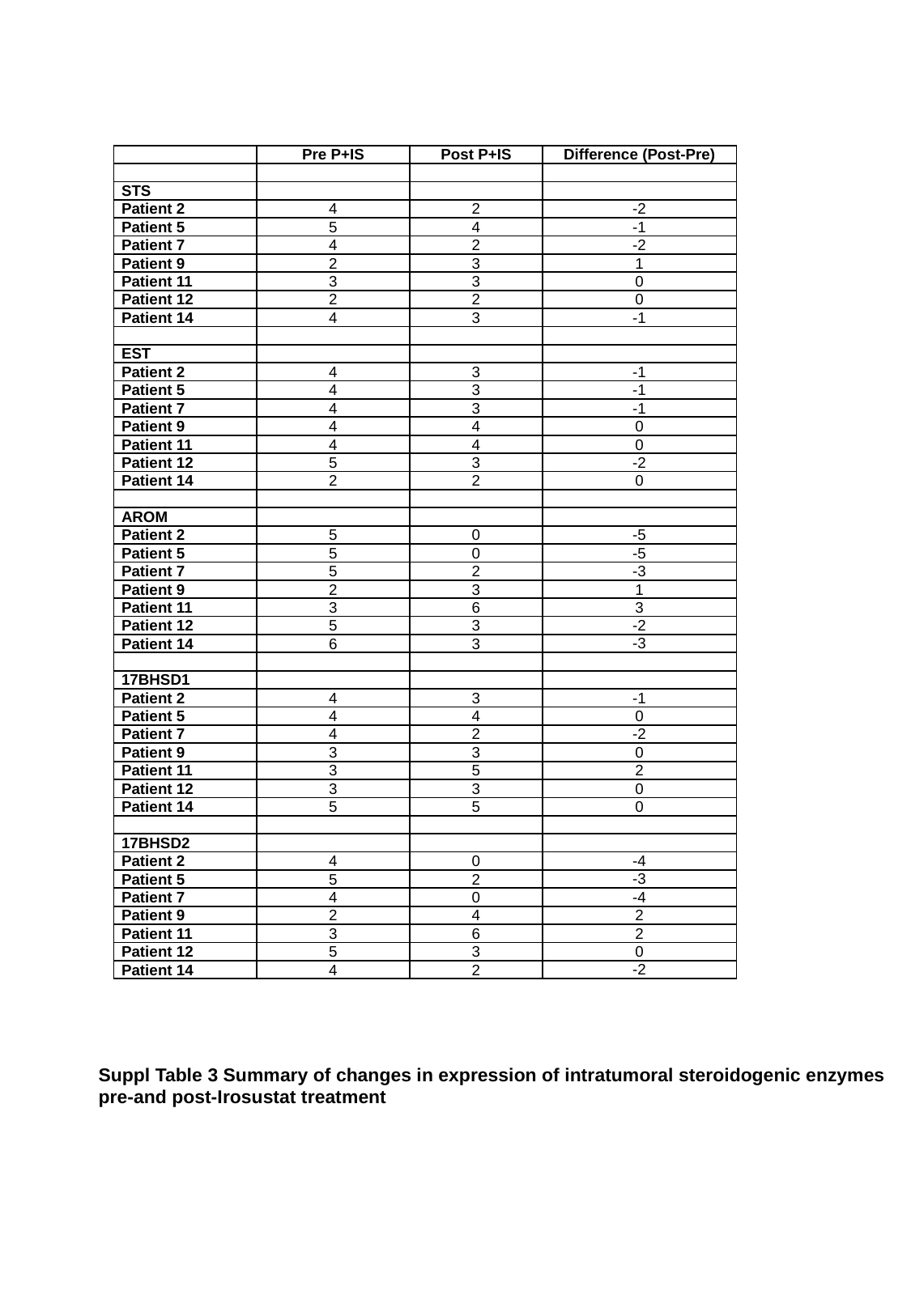

| | Pre P+IS | Post P+IS | Difference (Post-Pre) |
| --- | --- | --- | --- |
| | | | |
| STS | | | |
| Patient 2 | 4 | 2 | -2 |
| Patient 5 | 5 | 4 | -1 |
| Patient 7 | 4 | 2 | -2 |
| Patient 9 | 2 | 3 | 1 |
| Patient 11 | 3 | 3 | 0 |
| Patient 12 | 2 | 2 | 0 |
| Patient 14 | 4 | 3 | -1 |
| | | | |
| EST | | | |
| Patient 2 | 4 | 3 | -1 |
| Patient 5 | 4 | 3 | -1 |
| Patient 7 | 4 | 3 | -1 |
| Patient 9 | 4 | 4 | 0 |
| Patient 11 | 4 | 4 | 0 |
| Patient 12 | 5 | 3 | -2 |
| Patient 14 | 2 | 2 | 0 |
| | | | |
| AROM | | | |
| Patient 2 | 5 | 0 | -5 |
| Patient 5 | 5 | 0 | -5 |
| Patient 7 | 5 | 2 | -3 |
| Patient 9 | 2 | 3 | 1 |
| Patient 11 | 3 | 6 | 3 |
| Patient 12 | 5 | 3 | -2 |
| Patient 14 | 6 | 3 | -3 |
| | | | |
| 17BHSD1 | | | |
| Patient 2 | 4 | 3 | -1 |
| Patient 5 | 4 | 4 | 0 |
| Patient 7 | 4 | 2 | -2 |
| Patient 9 | 3 | 3 | 0 |
| Patient 11 | 3 | 5 | 2 |
| Patient 12 | 3 | 3 | 0 |
| Patient 14 | 5 | 5 | 0 |
| | | | |
| 17BHSD2 | | | |
| Patient 2 | 4 | 0 | -4 |
| Patient 5 | 5 | 2 | -3 |
| Patient 7 | 4 | 0 | -4 |
| Patient 9 | 2 | 4 | 2 |
| Patient 11 | 3 | 6 | 2 |
| Patient 12 | 5 | 3 | 0 |
| Patient 14 | 4 | 2 | -2 |
Suppl Table 3 Summary of changes in expression of intratumoral steroidogenic enzymes
pre-and post-Irosustat treatment

## Slide 5
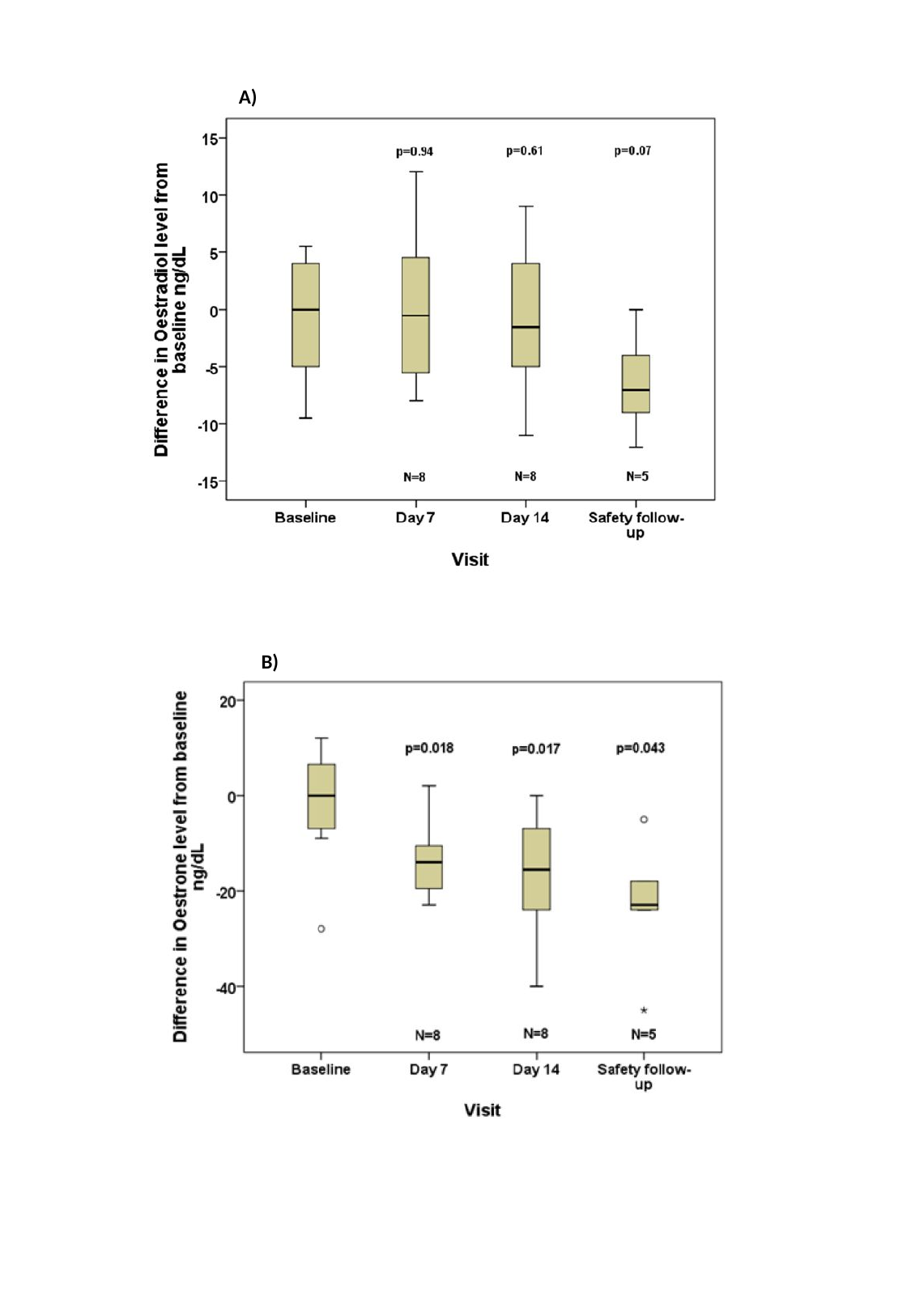

A)
B)

## Slide 6
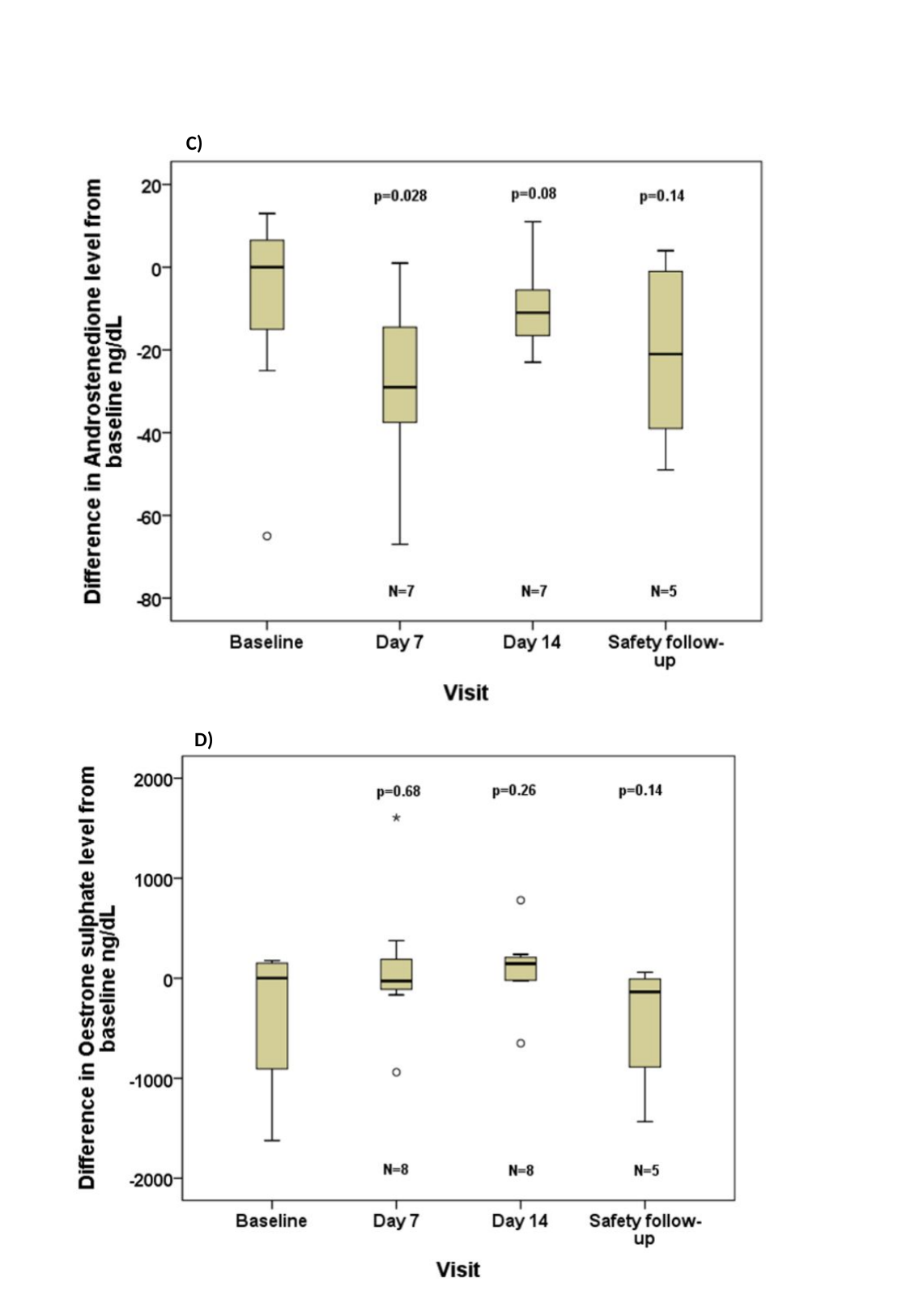

C)
D)

## Slide 7
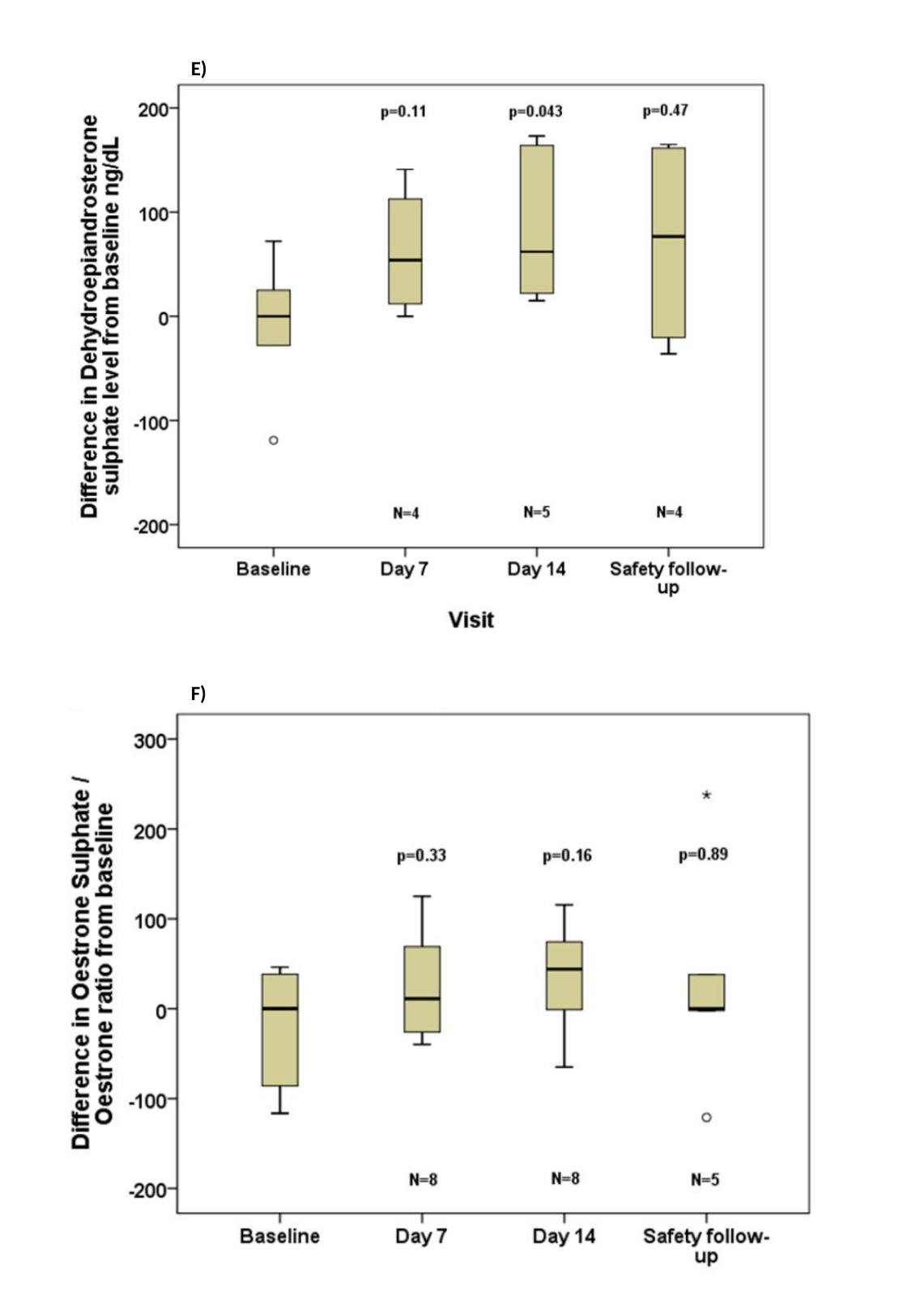

E)
F)

## Slide 8
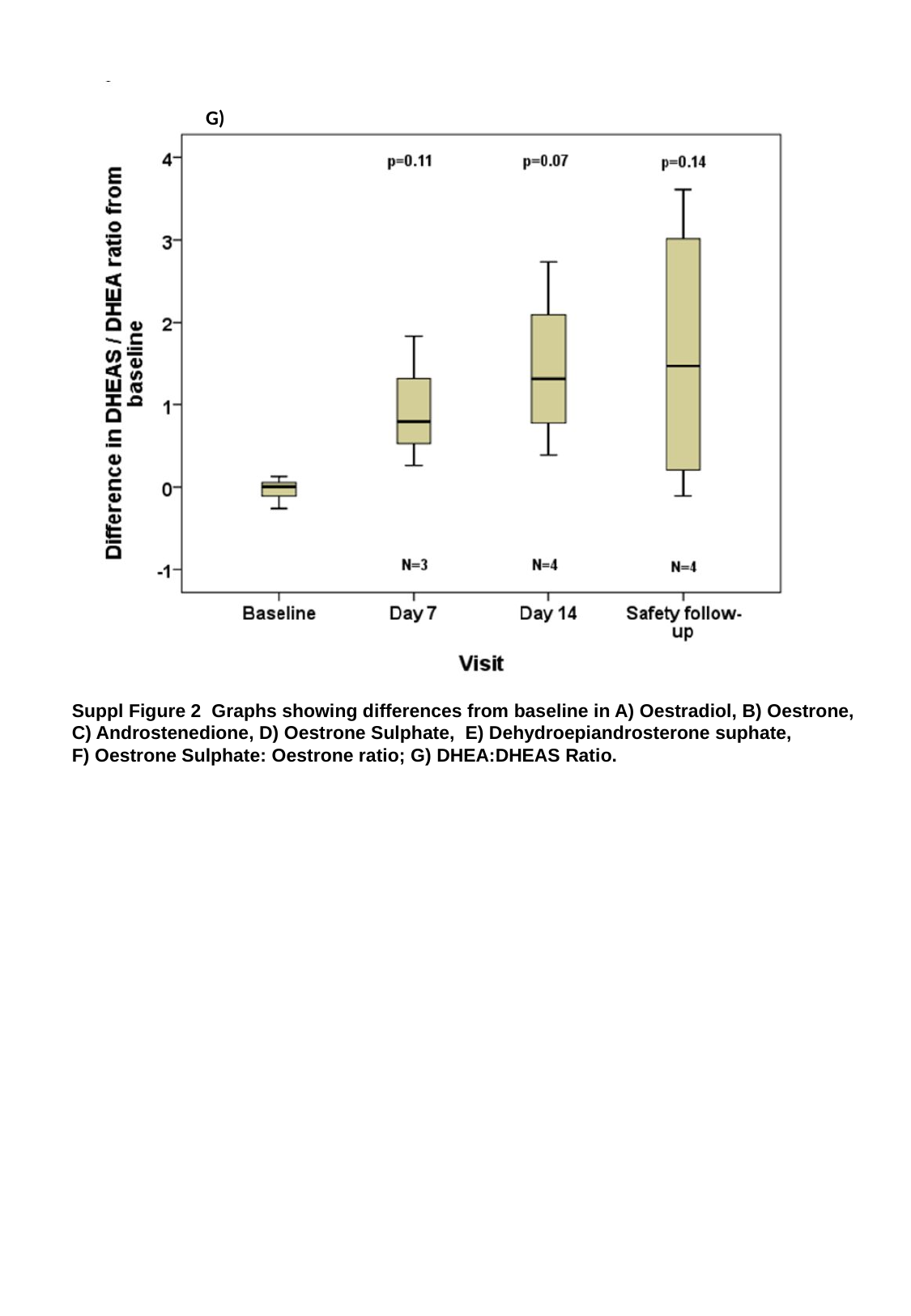

G)
Suppl Figure 2 Graphs showing differences from baseline in A) Oestradiol, B) Oestrone,
C) Androstenedione, D) Oestrone Sulphate, E) Dehydroepiandrosterone suphate,
F) Oestrone Sulphate: Oestrone ratio; G) DHEA:DHEAS Ratio.
